# Sex steroid hormones and brain function: PET imaging as a tool for research

**DOI:** 10.1111/jne.12565

**Published:** 2018-02-18

**Authors:** R. Moraga‐Amaro, A. van Waarde, J. Doorduin, E. F. J. de Vries

**Affiliations:** ^1^ Department of Nuclear Medicine and Molecular Imaging University Medical Center Groningen University of Groningen Groningen The Netherlands

**Keywords:** androgen receptor, neuroimaging, oestrogen receptor, positron emission tomography, sex steroid hormones

## Abstract

Sex steroid hormones are major regulators of sexual characteristic among species. These hormones, however, are also produced in the brain. Steroidal hormone‐mediated signalling via the corresponding hormone receptors can influence brain function at the cellular level and thus affect behaviour and higher brain functions. Altered steroid hormone signalling has been associated with psychiatric disorders, such as anxiety and depression. Neurosteroids are also considered to have a neuroprotective effect in neurodegenerative diseases. So far, the role of steroid hormone receptors in physiological and pathological conditions has mainly been investigated post mortem on animal or human brain tissues. To study the dynamic interplay between sex steroids, their receptors, brain function and behaviour in psychiatric and neurological disorders in a longitudinal manner, however, non‐invasive techniques are needed. Positron emission tomography (PET) is a non‐invasive imaging tool that is used to quantitatively investigate a variety of physiological and biochemical parameters in vivo. PET uses radiotracers aimed at a specific target (eg, receptor, enzyme, transporter) to visualise the processes of interest. In this review, we discuss the current status of the use of PET imaging for studying sex steroid hormones in the brain. So far, PET has mainly been investigated as a tool to measure (changes in) sex hormone receptor expression in the brain, to measure a key enzyme in the steroid synthesis pathway (aromatase) and to evaluate the effects of hormonal treatment by imaging specific downstream processes in the brain. Although validated radiotracers for a number of targets are still warranted, PET can already be a useful technique for steroid hormone research and facilitate the translation of interesting findings in animal studies to clinical trials in patients.

## 1 INTRODUCTION

**Figure 1:**
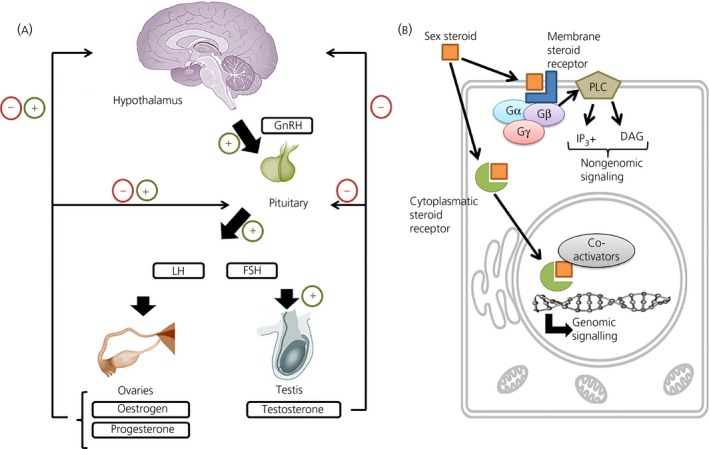
Effects of sex steroids at both physiological and cellular levels. (A) The regulatory processes for the synthesis of sex steroids by the hypothalamic‐pituitary‐gonadal (HPG) axis. The hypothalamus regulates the production of luteinising hormone (LH) and follicle‐stimulating hormone (FSH) via the release of gonadotrophin‐releasing hormone (GnRH). Both LH and FSH stimulate the synthesis and release of oestrogens and progesterone from the ovaries in females, as well as testosterone from the testis in males. At the same time, these sex steroids can regulate the release of GnRH from the hypothalamus, as well as LH and FSH from the pituitary. (B) General scheme of sex steroid effects at cellular level. Sex hormones can bind to either cytoplasmatic receptors or membrane‐associated receptors. When the molecules bind to membrane receptors, the receptor (coupled to G protein subunits complex: Gα, Gβ and Gγ) activates phospholipase C (PLC) to exert a rapid nongenomic responses via the second messengers inositol phosphate 3 (IP_3_+) and diacylglycerol (DAG). On the other hand, when they bind to cytoplasmatic receptors, the complex is translocated to the nucleus (with the help of different co‐activators) to exert genomic effects

Sex steroid hormones are a family of steroidal hormones that can be divided into 3 classes: oestrogens, progestins and androgens. These hormones are major regulators of sexual functions, including the reproductive cycle, reproductive physiology and the development of accessory reproductive organs.^1^ However, our vision of the function of these hormones has been expanded because they not only regulate sexual behaviour, but also affect brain functions, such as memory,^2^ anxiety‐related behaviour^3^ and other functions at the cellular level.^4^ Sex steroid hormones are mainly synthesised by the ovaries and testis. The hypothalamic‐pituitary‐gonadal (HPG) axis is the main system by which the production and release of sex steroids is regulated.^5^ Circulating sex hormones can stimulate the release of gonadothropin‐release hormones (GnRH) at the hypothalamus. GnRH induces the release of luteinising hormone (LH) and follicle‐stimulating hormone (FSH) in the pituitary, which activate the secretion of steroidal sex hormones from the gonads (Figure 1A). Peripheral sex hormones are present in the plasma, where they are mainly bound to plasma proteins such as sex hormone binding globulin (SHBG) or corticosteroid binding globulin (CBG).^6^ SHBG has high affinity for both oestrogens and androgens, whereas progesterone is bound by CBG. These globulins protect steroid hormones against metabolic degradation and, consequently, the fraction of free steroid hormones in plasma is small. Yet, this small fraction of unbound steroid hormones can readily cross the blood‐brain barrier by passive diffusion as a result of the lipophilic nature of steroids. However, there is also a significant contribution of de novo synthesised steroid hormones in the brain because the brain itself contains the enzymes needed for the synthesis of these steroids.^7^ Sex hormones produced in the brain include 17β‐oestradiol, testosterone and progesterone, along with other neuroactive steroids such as pregnenolone, dehydroepiandrosterone and allopregnanolone.^8^

In recent decades, the specific receptors for sex steroid hormones were found to be expressed in the brain.^9^ Currently, most information has been obtained from animal experiments, which cannot easily be translated to humans, as well as from post‐mortem analysis of human brain tissue.^10^, ^11^ In most studies, western blotting and in situ hybridisation have been used to quantify hormone receptors in the brain.^9^, ^12^ Such techniques would not allow research on the biology of steroid hormones and their receptors in the living human brain. One approach with respect to non‐invasively investigating sex hormone receptors in the brain is the use of positron emission tomography (PET) with radiolabelled receptor ligands. PET allows the quantification of functional parameters, such as receptor density and occupancy.^13^ PET imaging of steroid receptors is already widely used in oncology to visualise receptor expression and receptor occupancy in hormone‐sensitive tumours such as breast and prostate cancer.^14^ By contrast, sex hormone receptor imaging in the brain is still in its infancy.^15^ Sex steroid receptor imaging in neuroscience suffers from some additional hurdles, such as the low receptor expression in some brain regions^16^ and a poor penetration of radioligands through the blood‐brain barrier.

In this review, we survey the available literature about the use of PET imaging in the field of neuroendocrinology, in which imaging data are directly or indirectly correlated with sex steroid hormone (receptor) levels. We discuss the role of sex steroids in brain function and behaviour, give an overview of the tracers that are currently available for PET imaging of hormone receptors and their applicability in brain research, and summarise the results of PET imaging of the downstream effects of sex steroids in the brain. Based on these data, we propose that PET is a promising technique for future translational research in this field.

## 2 SEX STEROID HORMONES AND BRAIN FUNCTION

Oestrogens can exert their effects through either intracellular or membrane‐associated oestrogen receptors (ERs); in particular, the intracellular receptors ERα and ERβ, and membrane‐associated G‐protein regulator motifs. Upon binding of oestrogen to the ER, the ligand‐receptor complex dimerises and migrates to the nucleus, where the dimer can bind to hormone response elements (HRE) in the promotor region of oestrogen‐responsive genes. Activation of the HRE leads to the induction or the repression of gene transcription. In addition to this genomic signalling pathway, sex steroids can act via nongenomic signalling (Figure 1B) (for a review, see Kawata et al^17^). Oestrogen signalling can affect various aspects of brain function and behaviour. Most information about the relationship between oestrogens and brain disorders was obtained from studies in female animals or women demonstrating behavioural differences between the different stages of the menstrual cycle. There is ample evidence for a role of oestrogens in anxiety and depression, both from animals and humans.^18^ Women are vulnerable to depression when the concentration of sex hormones changes markedly. This can lead to pre‐menstrual dysphoric disorder, post‐partum depression and perimenopausal or postmenopausal depression.^19^ Oestrogens have antidepressant effects when they are administered either alone or in combination with antidepressants^20^, ^21^ and, consequently, oestrogen replacement therapy can be used to prevent the development of depression in individuals who are at risk.^22^ Oestrogens can also have neuroprotective effects. High levels of circulating oestrogens are associated with less ischaemia‐induced brain injury.^23^ A similar effect is also observed when high levels of endogenous oestrogens are synthesised in the brain.^24^ Oestrogens were found to play a role in neuronal plasticity and spine synapse formation.^25^, ^26^ Furthermore, many studies have shown positive effects of oestrogens on cognition.^27^, ^28^, ^29^ In Alzheimer's disease, oestrogens have been shown to protect neurones against the toxicity of amyloid plaques.^30^ Nevertheless, more studies are necessary^31^ because investigators from the Women's Health Initiative Memory Study found that therapy with a combination of oestrogen and progestin increased the risk for dementia in postmenopausal women and did not improve their performance in mild cognitive tasks.^32^ For this reason, the contribution of oestrogens and the molecular dynamics of their interaction with other hormones and neurotransmitters should be determined to obtain a better understanding of the role of these steroids in brain function and neuroprotection.

Progestins can exert their effects through both intracellular progestin receptors (PR‐A and PR‐B) and membrane‐associated PRs. In addition, these neuroactive steroids can also interact with several other receptors and ion channels.^33^ For example, several steroid hormones, including progesterone, were found to bind to sigma‐1 receptors.^34^ Progesterone can act as a sigma‐1 receptor antagonist.^35^ Under ischaemic conditions, progesterone antagonism of sigma‐1 receptors can be neuroprotective because it attenuates the NMDA‐induced influx of Ca^2+^ via the NMDA receptor ion channel.^36^ Progestins can also interact with oestrogens in the brain, such as in the regulation of synapse formation.^37^ Progestins are also involved in processes such as maintenance of the structural integrity of myelin,^38^ regulation of spinogenesis, synaptogenesis, neuronal survival and dendritic growth.^39^, ^40^, ^41^ There is evidence indicating that the administration of exogenous progesterone in animal models of traumatic brain injury and ischaemia can decrease the lesion volume in the brain^42^ and decrease cognitive deficits.^43^ Likewise, progestins can exhibit a neuroprotective effect in spinal cord injury.^44^ Evidence has also been presented suggesting a neuroprotective effect of progestins in other brain disorders, such as peripheral nerve injury, demyelinating disease, motoneurone diseases, seizures, depression and Alzheimer's disease.^18^, ^45^, ^46^, ^47^

Androgens exert their effects through the androgen receptor (AR) subtypes AR‐A and AR‐B. Androgens are known to affect various brain functions and behaviour. The most common behavioural role of androgens is related to aggression. An excess of circulating androgens induces aggressive behaviour in both males and females.^48^ Androgens are also involved in depression and anxiety‐like disorders, especially after menopause in women and during hypogonadism in men.^49^ Alterations of testosterone levels were associated with an increased risk of mood disorders and psychosis.^50^ Anabolic abuse and hyper‐ or hypoandrogenism are related to mood changes^51^ and the incidence of depression.^52^ On the other hand, androgens can also have a neuroprotective role. Long‐term exposure to androgens increases hippocampal neurogenesis and modulates the survival of new neurones.^53^ Androgens also play a role in synapse formation and they are capable of inducing the formation of spine synapses,^54^ which appears to be mediated by NMDA activity.^55^

## 3 PET TRACERS FOR STEROID HORMONE RECEPTORS IN BRAIN RESEARCH

Despite the increasing knowledge on the roles of sex steroid hormones, many aspects of the functions and mechanisms of action of sex steroid hormones in the brain are still incompletely understood and require further research. Non‐invasive imaging tools such as PET could facilitate such research. Several PET tracers for steroid hormone receptors are available and have been successfully used in oncology, although, to date, there are only few studies in which they have been used for brain research. Most studies with PET tracers for steroid hormone receptors use either autoradiography or ex vivo tissue counting. So far, only a few studies have measured the in vivo distribution of steroid receptor ligands in rodents, whereas imaging studies of the human brain are lacking.

The most frequently used PET tracer for imaging oestrogen receptors is 16α‐[^18^F] fluoro‐17ß‐oestradiol ([^18^F]FES). [^18^F]FES has been successfully used in both preclinical and clinical studies, mostly in breast cancer.^56^ [^18^F]FES was the first PET tracer to be applied for quantitative ex vivo assessment of oestrogen receptors in the brain. The brain of female rats was dissected and radioactivity in different brain areas was measured ex vivo with a γ counter. By applying different distribution times, information about the kinetics of the tracer in the rat brain was obtained.^16^ Specific binding of the tracer was observed only in brain regions with high ER density, such as the pituitary and hypothalamus. Specific binding could be quantified both by equilibrium and dynamic kinetic analysis.^16^ Two years later, [^18^F]FES PET was successfully used to identify ER expression in the tumour of six patients with brain meningiomas.^57^ Later studies, including our own, have shown that [^18^F]FES PET is able to detect ER‐expression in brain metastases of ER‐sensitive tumours such as breast cancer.

**Figure 2:**
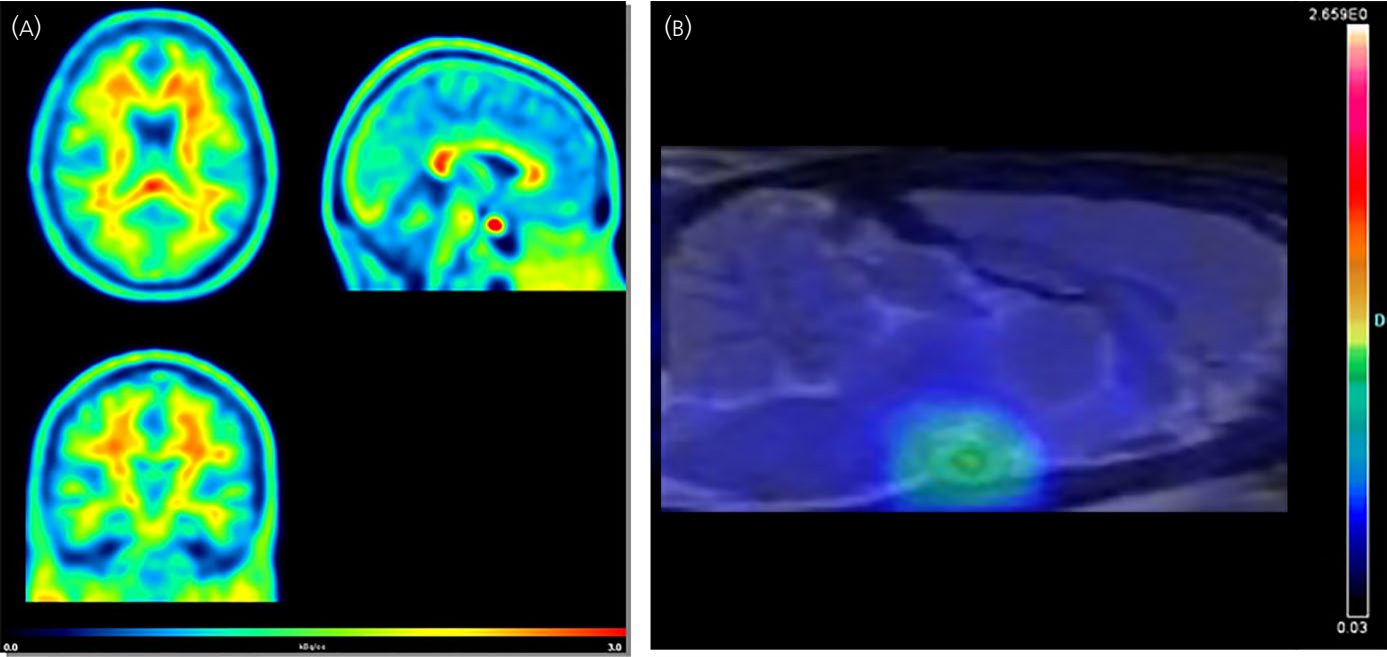
(A) Positron emission tomography (PET) images of the distribution of the oestrogen receptor tracer 16α‐[^18^F] fluoro‐17ß‐oestradiol ([^18^F]FES) in the brain of a healthy postmenopausal woman. The images were acquired 60‐90 min after injection of 200 MBq of [^18^F]FES. Tracer uptake is presented as kBq/cc images. The pituitary is clearly visible as a hotspot (red) in the sagittal image (top right). In addition, the images mainly show uptake in white matter. (B) PET scan of a naïve female rat brain, 60‐90 min after injection of 25 MBq of [^18^F]FES. The PET scan is co‐registered with a magnetic resonance imaging template of the brain to provide an anatomical reference. The highlighted spot represents the activity of the tracer in the pituitary/hypothalamus

More than a decade after the experiments of Moresco et al,^16^ our group investigated whether ER in the rat brain could be quantified in vivo using [^18^F]FES with a dedicated small‐animal PET scanner.^58^ The results obtained were in agreement with the ex vivo data of Moresco et al.^16^ Specific binding was observed in the pituitary and hypothalamus, which are both brain regions with a high ER density, but not in other parts of the brain.^58^ Ovariectomy resulted in an increase in tracer uptake in the pituitary and hypothalamus, whereas administration of exogenous oestradiol decreased [^18^F]FES uptake in these regions, indicating that tracer uptake was sensitive to circulating oestrogens competing for the binding site of the ER. Driven by the promising results in rats, we performed a small [^18^F]FES PET study in healthy volunteers. In postmenopausal women, [^18^F]FES PET showed a significantly higher tracer uptake in the pituitary compared to any other brain region, with only slight differences in [^18^F]FES uptake between these brain regions. Besides the pituitary, [^18^F]FES mainly accumulated in white matter (Figure 2). Administration of an experimental ER antagonist significantly reduced [^18^F]FES in the pituitary but not in any other brain region. [^18^F]FES uptake in white matter was also unaffected by the antagonist, suggesting that white matter uptake was dominated by nonspecific binding of the lipophilic tracer. [^18^F]FES PET could be applied to assess ER receptor occupancy of this experimental drug in pituitary^59^. By contrast to the aforementioned of PET studies in rats, [^18^F]FES did not show any specific binding in the hypothalamus in humans. This discrepancy might be related to species differences in receptor expression, nonspecific binding, plasma levels of SHBG or blood‐brain barrier penetration of the tracer. However, there is no concrete evidence available yet for any of these hypotheses.

Consequently, [^18^F]FES PET could be useful for assessing ER density in brain regions with high ER expression (pituitary), although it does not appear to be sufficiently sensitive for evaluating ER density in other brain regions. Therefore, the development of novel PET tracers with higher affinity is urgently warranted to boost the research in this area.

Some PET tracers have been developed for imaging of progestin receptors as well. 21‐[^18^F]Fluoro‐16α‐ethyl‐19‐norprogesterone ([^18^F]FENP) was evaluated as candidate tracer for the progestin receptor and initially appeared to show specific uptake in the uterus and in tumours of both rats and humans.^60^ However, further studies in cancer patients revealed that [^18^F]FENP could not detect the PR in a large fraction of positive tumours. [^18^F]FENP uptake did not correlate with PR levels and the tumour‐to‐background ratio was low.^61^ Moreover, the tracer was rapidly metabolised not only in the liver and blood, but also by tumour cells.^62^ Subsequently, tracers with increased metabolic stability have been developed and tested for imaging of PR in human breast cancer, including 21‐[^18^F]fluoro‐16α,17α‐[*(R)*‐(1′‐α‐furylmethylidene)‐dioxy]‐19‐norpregn‐4‐ene‐3,20‐dione ([^18^F]FFNP)^63^ and 4‐[^18^F]fluoropropyl‐tanaproget ([^18^F]FPTP).^64^ Both tracers showed specific uptake in the uterus, although, after the initial reports, no further studies have been published and none of the tracers has yet been tested for imaging of PR in the brain.

Several promising PET tracers for androgen receptors have been developed, especially for imaging of prostate cancer. The first tracer for PET imaging of the androgen receptors was 20‐[^18^F]fluoromibolerone ([^18^F]Fmib), which was tested in both rats and baboons with promising results.^65^, ^66^ More tracers have been synthesised and tested as markers of prostate cancer,^67^ of which 16ß‐[^18^F]fluorodihydrotestosterone ([^18^F]FDHT) is the most promising so far. [^18^F]FDHT was successfully applied to image the expression of AR in tumours not only in preclinical studies, but also in patients with prostate cancer.^68^ Our group has tested [^18^F]FDHT for PET imaging of AR in the rat brain.^69^ Our study showed that [^18^F]FDHT is metabolised very rapidly in rats, and its uptake in the brain is very low.^69^ This results in a poor signal‐to‐noise ratio, which precludes the detection of AR in the rat brain. By contrast to rats, humans express SHBG, which can protect steroids such as [^18^F]FDHT from metabolic degradation.^70^ Despite the disappointing results obtained in rats, the stabilising effect of SHBG in men would still warrant investigation of the ability of [^18^F]FDHT PET to visualise AR receptors in the brain of humans.

In conclusion, it can be proposed that PET has potential as a non‐invasive tool for assessing the expression of steroid receptors in the brain, provided tracers become available that can penetrate the blood‐brain barrier and have higher affinity and metabolic stability.

## 4 PET IMAGING OF AROMATASE AS A BIOMARKER FOR OESTROGEN SYNTHESIS

Aromatase is a key enzyme in the biosynthesis of oestrogens; it catalyses the conversion of testosterone into oestradiol.^71^ Aromatase is expressed in a wide variety of tissues, including ovaries, adipose tissue, skin, testicles, muscle, liver and the central nervous system. Aromatase has been suggested as a biomarker for neuroprotection because it increases the local levels of oestrogens in injured neurones in the brain.^72^ Aromatase is not expressed constitutively in the brain but can be induced by testosterone or dihydrotestosterone.^73^ Brain aromatase is involved in, amongst others, the regulation of sexual behaviour, emotional behaviour, aggression, cognition, memory and neuroprotection,^73^ making this enzyme an interesting target for the study of sex steroid hormones in the brain.

Tracers for aromatase are generally based on enzyme inhibitors. The nonsteroidal aromatase inhibitor [^11^C]vorozole is the most tested tracer in this field. PET imaging studies with [^11^C]vorozole in rhesus monkeys showed specific binding to aromatase ex vivo in the medial amygdala, bed nucleus stria terminalis and the pre‐optic area, whereas in vivo binding only occurred in the medial amygdala and the pre‐optic area. Specific tracer uptake could be quantified by pharmacokinetic modelling, using cerebellum as a reference tissue.^74^ The same group subsequently applied this tracer in a nonhuman primate and a rodent model of anabolic steroid abuse.^75^ PET with [^11^C]vorozole demonstrated increased aromatase levels in the bed nucleus of the stria terminalis and the pre‐optic areas of rats treated with anabolic androgenic steroids, as well as in the hypothalamus of macaque monkeys treated with these steroids.^76^, ^77^ [^11^C]vorozole PET imaging in baboons showed that the menstruation cycle had a significant effect on tracer binding in the brain.^78^ The first human PET study with [^11^C]vorozole was performed in 2010,^79^ demonstrating the specificity and kinetics of this tracer in the human brain. This study was followed by recent studies evaluating the radiation dosimetry and binding kinetics of [^11^C]vorozole in healthy men and women.^80^, ^81^

Besides [^11^C]vorozole, two other tracers for the assessment of brain aromatase were tested. [^11^C]Letrozole was investigated in baboons and it was concluded that this tracer is not suitable for brain research because of the absence of regional specific, saturable binding in the brain.^82^ A later study tested the tracer [^11^C]cetrozole in rhesus monkeys. In this subsequent study, [^11^C]cetrozole displayed better selectivity and specificity, as well as a higher signal‐to‐noise ratio, than [^11^C]vorozole. Therefore, it was better suited for the quantitative analysis of aromatase expression in the amygdala, hypothalamus and nucleus accumbens in monkeys.^83^ So far, however, no studies on the use of [^11^C]cetrozole PET in humans have been published.

**Figure 3:**
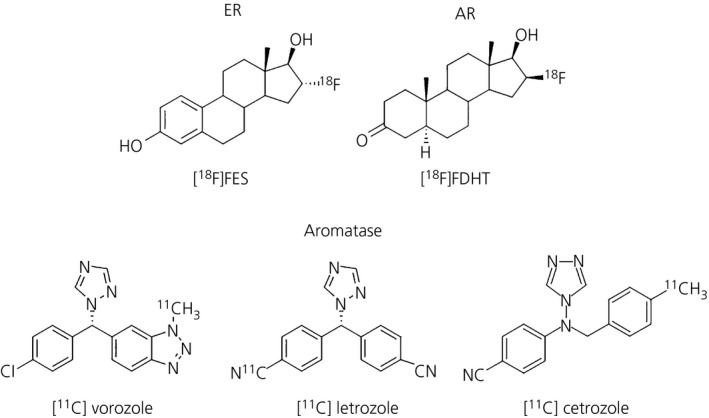
Chemical structure of tested radiotracers in the brain for both sex hormone receptors and oestrogen synthesis. 16α‐[^18^F] fluoro‐17ß‐oestradiol ([^18^F]FES) is the radioligand used for oestrogen receptors (ER). 16ß‐[^18^F]fluorodihydrotestosterone ([^18^F]FDHT) corresponds to the tracer tested in the rat brain to visualise androgen receptors (AR). [^11^C]vorozole, [^11^C]letrozole and [^11^C]cetrozole are all tracers for aromatase quantification, which is the enzyme responsible for oestrogen production using testosterone as substrate

Accordingly, 2 suitable PET tracers for imaging of aromatase expression in animals have recently become available and one of them has been successfully evaluated in healthy volunteers. Further evaluation in clinical studies to obtain more insight regarding the value of these tracers is required. If successful, this PET imaging could provide a new impetus for clinical trials on the role of aromatase in health and disease. The chemical structure of radiotracers for sex hormone receptors and aromatase tested in the brain, is shown in Figure 3.

## 5 USE OF PET TRACERS TO STUDY SEX STEROID HORMONE‐INDUCED CHANGES IN BRAIN FUNCTION

PET imaging using radioligands of receptors related to sex steroid hormone signalling may provide valuable information about the interaction of these hormones with other signalling systems in the brain, as well as the possible behavioural outcome of that interaction, thus offering a wide range of possible studies. PET imaging may also be used to study the impact of steroid hormones on physiological or metabolic biomarkers. Below, we discuss some studies in which the downstream effects of hormonal changes were evaluated by PET imaging.

### 5.1 Impact of sex steroid hormones in cerebral blood flow

A general approach for studying the impact of steroid hormones is to detect activation of specific brain regions by measurement of the regional cerebral blood flow (rCBF). Changes in cerebral blood flow can be detected with PET using the tracer [^15^O]H_2_O.^84^, ^85^ Such rCBF changes can be related to specific changes in the physiological concentrations of sex hormones.

Only a few studies have applied [^15^O]H_2_O PET to study the impact of hormonal changes on brain activity so far. [^15^O]H_2_O PET has been used to investigate the effect of oestrogen replacement therapy on the rCBF. Significant longitudinal differences were found in rCBF activation patterns during cognitive tasks between controls and women on oestrogen replacement therapy.^84^, ^85^ In particular, oestrogen replacement users showed higher rCBF in the memory circuit hippocampus‐parahippocampal gyrus‐temporal lobe than non‐users. In another study, [^15^O]H_2_O PET was used to investigate the effect of endogenous testosterone on the rCBF in elderly men.^86^ Higher endogenous testosterone concentrations were found to correlate with a higher rCBF in brain regions associated with memory and attention. Recently, [^15^O]H_2_O PET was used to investigate the correlation between a decreased production of progesterone and oestradiol by the ovaries and hippocampal working memory, although no statistically significant changes in blood flow were found.^87^

Thus, the limited number of available studies suggests that measurement of the rCBF with [^15^O]H_2_O PET could be a useful tool for investigating the impact of sex steroid hormones on cognition. However, a disadvantage of [^15^O]H_2_O PET is the exposure of subjects to a radioactive substance. For many research questions, magnetic resonance imaging (MRI) techniques have now replaced [^15^O]H_2_O PET. Functional MRI using blood‐oxygen‐level dependent (BOLD) imaging is a frequently used technique for measuring regional brain activity. Although the BOLD signal is dependent on blood flow, it is not a direct measure of the rCBF. Functional MRI using arterial spin labelling (ALS), on the other hand, measures the transit of magnetically labelled water and thus can provide a direct measure of the rCBF. In many situations, ALS can therefore provide a suitable alternative for [^15^O]H_2_O PET.

### 5.2 Brain metabolism and sex hormones: [^18^F]FDG

2‐Deoxy‐2‐[^18^F]fluoro‐d‐glucose ([^18^F]FDG) is the most frequently used radiotracer in PET imaging. Because the metabolic properties of [^18^F]FDG are similar to those of d‐glucose, [^18^F]FDG PET can be used to detect tissues with changed glucose metabolism. [^18^F]FDG is mainly used for the diagnosis of cancer, infections and cardiovascular diseases.^88^ Glucose is the primary source of energy for the brain and, consequently, [^18^F]FDG PET can also be used to assess brain glucose metabolism, which is often used as a surrogate marker for brain activity. Thus, [^18^F]FDG PET can be used to assess changes in cerebral activity of specific brain areas during the time course of diseases and to evaluate the effect of treatment.

Few preclinical studies using [^18^F]FDG PET have been performed to investigate the effect of sex steroid hormones on brain glucose metabolism. The first study in this specific field aimed to determine the neural correlates of sexual competition in male rhesus macaques. The study showed metabolic differences between male monkeys confronted with threats to their exclusive sexual access to a female mate and controls. The differences in brain glucose metabolism were correlated with differences in testosterone levels.^89^ [^18^F]FDG PET studies in a rat model of traumatic brain injury aimed to visualise the effects of hormone therapy, using either synthetic or endogenous oestrogens.^90^, ^91^ These studies demonstrated that the steroid hormones reduced the trauma‐induced decrease in glucose metabolism, suggesting a beneficial effect on cellular survival.

[^18^F]FDG PET was also used in several human studies to investigate the effect of steroid hormones on brain metabolism. Reiman et al^92^ measured [^18^F]FDG uptake in specific brain regions of female volunteers aiming to study the effect of circulating oestrogens on brain glucose metabolism. Different [^18^F]FDG distribution patterns were observed during the different phases of the menstrual cycle. In particular, a higher [^18^F]FDG uptake was observed in thalamic, prefrontal, temporoparietal and inferior temporal regions during the mid‐follicular phase, whereas the mid‐luteal phase was associated with higher [^18^F]FDG uptake in superior temporal, anterior temporal, occipital, cerebellar, cingulate and anterior insular regions. Other studies investigated the effect of hormonal therapy or hormonal administration on brain metabolism. A useful approach for an assessment of the impact of steroid hormones on the metabolism of the human brain is the use of postmenopausal women or hypogonadal males because of the significant decrease in basal levels of sexual hormones in the body of these subjects. Eberling et al^93^ studied the effect of oestrogen agonists and antagonists in postmenopausal healthy women, or women with breast cancer. Changes in glucose metabolism were mainly observed in the frontal lobe and the hippocampus. A small [^18^F]FDG PET study in men with hypogonadism investigated the effect of testosterone substitution therapy on brain glucose metabolism during a mental rotation test.^94^ In 4 out of 6 subjects, testosterone substitution improved the mental rotation score and only these subjects showed an increase in [^18^F]FDG uptake in one specific brain area compared to baseline. Remarkably, each of these individuals revealed a different area with enhanced tracer uptake (right inferior occipital gyrus, right inferior frontal gyrus, right middle temporal gyrus, left primary visual cortex), which makes it difficult to draw any conclusions.

Besides the investigation of regional glucose metabolism, it is possible to study the connectivity and network changes associated with steroid hormone treatment using [^18^F]FDG PET. Ottowitz et al^95^ investigated whether the connectivity of specific brain areas was associated with systemic hormone levels. They observed that oestradiol injections induced significant changes in [^18^F]FDG uptake and prefrontal‐hippocampal connectivity in postmenopausal women. When pre‐ and postmenopausal subjects were compared, changes in the amygdala‐cortical network connectivity were observed as well.^96^

**Figure 4:**
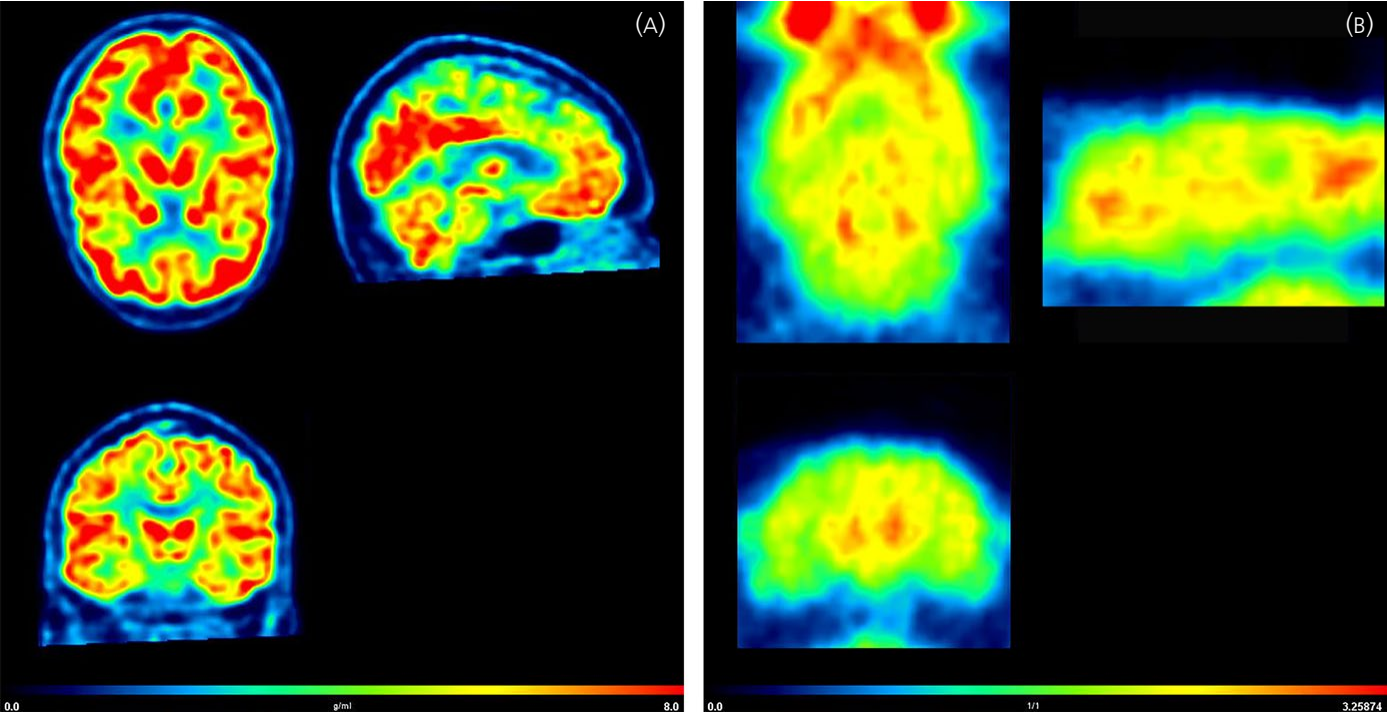
(A) 2‐Deoxy‐2‐[^18^F]fluoro‐d‐glucose ([^18^F]FDG) positron emission tomography (PET) images of the brain of a healthy woman. The subject had fasted for 6 hours prior to the scan. Static PET images were acquired 30‐35 min after injection of 200 MBq of [^18^F]FDG. [^18^F]FDG uptake is presented as standardised uptake values (SUV) and is a surrogate marker for brain glucose metabolism. (B) [^18^F]FDG PET images of the brain of a healthy female Wistar rat. The images were reconstructed from a 30‐min static scan that was acquired 45 min after i.v. injection of 17 MBq of [^18^F]FDG. [^18^F]FDG uptake is presented as the SUV

[^18^F]FDG PET can also be used to study secondary effects associated to hormonal therapies. [^18^F]FDG PET was applied to investigate possible neurobiological factors underlying the hot flashes as a secondary effect of hormone adjuvant treatment in breast cancer patients. Reduced glucose metabolism in the hypothalamus and insular cortex was found to be a predictor of the development of hot flashes.^97^ Other studies investigated the risk of developing neurocognitive disorders by hormone therapy in menopausal and postmenopausal women. Silverman et al^98^ investigated the effect of oestrogen‐containing hormone therapies on brain glucose metabolism in postmenopausal women at risk of Alzheimer's disease. [^18^F]FDG PET demonstrated that oestrogens had a neuroprotective effect, which was associated with a better score on verbal memory. Women continuing on oestrogen‐based hormone therapy showed a preservation of glucose metabolism in the precuneus/posterior cingulate cortical area, comprising a brain region known to show significant degeneration in the early stages of Alzheimer's disease.^99^ [^18^F]FDG PET has also been used to study the correlation between brain metabolism and oestradiol brain levels in postmenopausal women with Alzheimer's disease. In a small study, a direct linear correlation was found between hippocampal glucose metabolism and oestradiol levels in the cerebrospinal fluid.^100^ [^18^F]FDG PET was also able to reveal regional changes in brain glucose metabolism as a result of testosterone replacement therapy in two hypogonadal patients with Alzheimer's disease.^101^ Furthermore, [^18^F]FDG PET was able to demonstrate a compensatory effect of testosterone administration on brain hypometabolism in women with anorexia nervosa.^102^ An example of [^18^F]FDG imaging in the brain is provided in Figure 4.

### 5.3 Sex steroid hormones and neurotransmitter activity regulation

Sex steroid hormones are known to participate in many developmental and regulatory processes in the brain. Most of these effects are mediated by either direct actions on hormone receptors or by indirect modulation of other neurotransmitter systems.^103^

Serotonin is an important neurotransmitter that plays a central role in brain development, stress reactivity, mood and several psychiatric disorders.^104^ Serotonin signalling can be affected by sex steroid hormones.^103^ Serotonin receptors (5‐HTR) are part of a complex signalling pathway in the brain and can be divided into 7 different families, each with different subtypes. PET tracers are available for several 5‐HTR subtypes. So far, only few 5‐HTR tracers have been used to study the interactions between sex hormones and serotonin neurotransmission.

[^11^C]‐WAY100635 can be used to measure the expression of the 5‐HTR_1A_ subtype. Some studies have used this tracer to assess possible correlations between 5HTR_1A_ expression and circulating hormone concentrations in humans. PET imaging with [^11^C]‐WAY100635 showed that 5‐HTR_1A_ expression in the hippocampus of healthy women is positively correlated with levels of the androgen and oestrogen precursor dehydroepiandrosterone (DHEA), suggesting a role for the serotoninergic system in the up‐regulation of sex steroids.^105^ PET also showed that lateralisation of 5‐HTR_1A_ in language areas (hemispheric asymmetry) is positively correlated with plasma levels of sex hormones.^106^ Other PET studies with [^11^C]‐WAY100635 have shown that increased 5HTR_1A_ expression was associated with enhanced progesterone and DHEA levels in pre‐ and postmenopausal women.^107^ Sex hormone levels were found to be correlated with test scores for aggression and 5‐HTR_1A_ tracer uptake in frontal areas.^108^ Serotonin changes have also been studied in the brain of menopausal women treated with hormone therapy, but no significant differences in [^11^C]‐WAY100635 uptake were found between subjects treated with oestradiol alone or oestradiol + progesterone.^109^

Another receptor of interest is the 5‐HTR_2A_. Longitudinal PET studies with the tracer [^18^F]altanserin showed increased 5‐HTR_2A_ binding in the whole brain and in specific brain regions (eg, the hypothalamus and cortex) of postmenopausal women that were first treated with oestradiol alone, and were later treated with the combination of oestradiol with progesterone.^110^, ^111^ Another study using the same radiotracer showed a positive correlation between cortical [^18^F]altanserin binding and levels of endogenous oestradiol in men.^112^

A recent study used [^11^C]SB207145, a specific tracer for imaging of 5‐HTR_4_. A negative correlation was observed between binding of [^11^C]SB207145 to 5‐HTR_4_ receptors in the whole brain and both oestradiol and testosterone levels in healthy men.^113^ Several studies used the radiolabelled serotonin precursor [^11^C]‐5‐hydroxytryptophan ([^11^C]‐5‐HTP) and tracers for the serotonin transporter (SERT). [^11^C]5‐HTP and [^15^O]H_2_O were used to investigate the correlation between regional serotonin synthesis, blood flow and the levels of sex hormones and symptoms of premenstrual dysphoria in women, showing an inverse correlation between menstrual phase changes in plasma oestradiol levels and changes in the right‐to‐left [^11^C]5‐HTP uptake ratios in the dorsolateral prefrontal cortex.^114^ Two PET imaging studies with different tracers examined the interplay between sex hormones and the expression of SERT, which is known to be related to brain processes affected in psychiatric disorders.^115^ Frokjaer et al^116^ investigated the influence of therapy with GnRH agonists on depressive symptoms and SERT availability using PET with the tracer [^11^C]DASB. GnRH therapy decreased oestrogen levels, induced depressive symptoms and increased SERT availability in neocortex. Jovanovic et al^117^ used [^11^C]MADAM PET and showed a decrease in SERT expression after long‐term treatment of postmenopausal women with oestrogen alone or a combination of oestrogen and testosterone.

A small study with the dopamine receptor ligand [^11^C]raclopride aimed to visualise changes in dopamine D2/D3 receptor availability over the different phases of the menstrual cycle in healthy women. However, no significant changes in [^11^C]raclopride binding were observed.^118^ Another study evaluated the effect of circulating testosterone and oestradiol on D2/D3 receptor availability in Göttingen minipigs treated with chronic amphetamine in a longitudinal design, using [^11^C]raclopride PET. The study did not reveal any significant correlation between the imaging results and the plasma concentrations of testosterone or oestradiol either.^119^ A later study used a different tracer for the same receptor ([^18^F]fluoroclebopride) in female cynomolgus monkeys and demonstrated differences in the distribution volume ratio in the caudate nucleus and the putamen between the luteal and follicular phase.^120^ Two additional studies on the dopaminergic system have been reported upon. Kindlundh et al^121^ used three different tracers to measure dopaminergic changes in the rat brain as a result of treatment with anabolic‐androgenic steroids. Changes in the density of dopamine transporters were assessed using [^11^C]FE‐β‐CIT, changes in the density of D1 receptors were assessed with [^11^C]‐(+)‐SCH23390 and changes in D2/D3 receptors were assessed with [^11^C]raclopride. Treatment with the androgen nandrolone only caused an increased in [^11^C]FE‐β‐CIT binding in striatum, indicative of up‐regulation of dopamine transporters.^121^ Dopamine receptor availability was not affected. Another study assessed the effect of steroid hormones on dopamine metabolism in ovariectomised female rhesus monkeys, using the tracer [^18^F]6‐fluoro‐l‐*m*‐tyrosine. However, significant changes in the concentrations of the dopamine precursor were not detected.^122^

Interactions of steroid hormones with the GABAergic neurotansmitter systems have been studied using PET with [^18^F]flumazenil, a tracer for GABA_A_ receptors. The effect of ovariectomy and oestradiol replacement on GABA_A_ receptor expression was investigated in a social subordination model of female rhesus monkeys. Ovariectomy caused an increase in [^18^F]flumazenil binding in the cortex and other brain areas. This effect was reversed by hormone replacement therapy.^123^

The aforementioned studies show that sex steroid hormones can have an effect on brain neurotransmitter systems and also that these effects can be monitored non‐invasively with PET. So far, only few publication describe the use of this approach in neuroendocrinology studies, indicating an area of research that still remains unexplored. Most of the studies showed interactions of sex hormones with major neurotransmitter systems involved in psychiatric disorders such as serotonin and dopamine, positioning them as likely targets for future research in this field.

## 6 CONCLUSIONS

There is ongoing research on the influence of sex steroid hormones on brain development and brain function. Although the expression of sex steroid hormone receptors in the brain has been demonstrated^9^ and some roles of these hormones in the brain have been elucidated,^2^, ^3^, ^4^ there is still a large gap in our knowledge of these hormone system. This can be partly be ascribed to the lack of suitable techniques available for assessing the dynamics and interplay of these molecules in the living brain. Non‐invasive imaging could offer a good opportunity to investigate the role of sex steroid hormones and their receptors in the brain in health and disease.

Specific radiotracers for PET imaging of oestrogen, progestin and androgen receptors have been developed, although only few of them have been tested in the brain. Some successful studies, especially using the ER tracer [^18^F]FES, have been performed, although low uptake in brain areas with low receptor density, rapid tracer metabolism and unfavourable kinetics of many tracers limit the application of these tracers for the visualisation and quantification of changes of steroid receptor density in specific brain areas. Tracers with higher affinities and metabolic stability and better blood‐brain barrier penetration are needed to expand this research field.

PET imaging can also be used to quantify the effects of sex steroids on brain perfusion and metabolism. Hormone treatment in conditions such as menopause, hypogonadism and steroid abuse appears to provide useful paradigms for studying the effect of steroid signalling on brain activity or examining the relationship between stress hormone levels and biological outcomes in humans. Studies of the correlations between hormone levels in plasma and regional tracer uptake may also provide useful information on the involvement of specific brain regions and regional connectivity. The use of animal models may also be useful because many experimental manipulations can be applied in animals but not in humans.

Furthermore, a plethora of PET tracers for specific neurotransmitter receptors and transporters are available. These tracers enable the investigation of the interaction between sex steroid hormones and various neurotransmitter systems. These studies could help to unravel the mechanisms that are responsible for the impact of sex steroid hormones on brain function and neuroprotection. An improved understanding of these effects could result in the improvement of existing hormone therapies. Studies could focus on, for example, discrimination of specific receptor functions in terms of fast and slow effects, sex differences and the mechanisms of action of steroids in diseases of the brain. We have reviewed PET studies related to the function of sex hormones in the brain. If the limitations identified can be overcome, PET may prove to be a promising non‐invasive technique that can be applied in both experimental animals and human subjects, which would facilitate the translation of interesting findings from studies in experimental animals into clinical trials in humans.

## CONFLICT OF INTERESTS

The authors declare that they have no conflicts of interest.

## AUTHOR CONTRIBUTIONS

RM, EdV, AvW and JD co‐wrote the manuscript. RM collated the material.
